# Assessment of the Botanical Origin of Polish Honeys Based on Physicochemical Properties and Bioactive Components with Chemometric Analysis

**DOI:** 10.3390/molecules26164801

**Published:** 2021-08-08

**Authors:** Maria Tarapatskyy, Patrycja Sowa, Grzegorz Zaguła, Małgorzata Dżugan, Czesław Puchalski

**Affiliations:** 1 Department of Bioenergetics, Food Analysis and Microbiology, Institute of Food Technology and Nutrition, College of Natural Sciences, University of Rzeszow, 35-601 Rzeszow, Poland; psowa@ur.edu.pl (P.S.); g_zagula@ur.edu.pl (G.Z.); cpuchal@ur.edu.pl (C.P.); 2 Department of Chemistry and Food Toxicology, Institute of Food Technology and Nutrition, College of Natural Sciences, University of Rzeszow, 35-601 Rzeszow, Poland; mdzugan@ur.edu.pl

**Keywords:** honey, bioactive compounds, proline, minerals, chemometrics

## Abstract

Is it possible to characterize the types of honey based on their chemical composition, their content of bioactive substances, and their physicochemical properties? The objective of this study was a comparative analysis of four types of honey from the Carpathian Foothills area, located in south-east Poland, based on the content of the main phenolic acids and proline, the mineral composition, and selected physicochemical properties. Most analyses, such as those of phenolic acids, sugars, and proline content, in honey samples were performed using chromatographic methods. These experiments demonstrated that honeydew honeys were the richest in phenolic acids, minerals, as well as oligosaccharides, compared to other honeys. Dark-colored honeys were characterized by the highest proline content. The dominant elements in all types of honey were potassium and calcium. The results of the present study show that analyses of specific phenolic acids, minerals, proline, and sugar content, in combination with chemometrics analysis, may successfully differentiate between the biological origins of honey samples and allow the preliminary verification of the samples before performing time-consuming pollen analysis.

## 1. Introduction

Honey is a food product made by honey bees *Apis mellifera* from nectar, honeydew, or both. For this reason, honey can be divided into honeydew and nectar honeys. Although honeydew honeys are produced by bees from the secretions of insects sucking on the plants, nectar honeys consist of the nectar of secretions of flowers or secretions from other live parts of plants [1]. Different physical parameters, such as the color, pH, conductivity, and even taste and aroma of honeys differ depending on the type of nectar flow/pollen collected by the bees, as well as their geographic origin [2,3]. The botanical origin of honey is one of its main quality parameters and its price is very often related to this origin [4]. Like its physical properties, the chemical composition of honeys is highly diverse and depends largely on the plant species from which the nectar or honeydew originates, as well as the bee species, geographic area, season, state of the environment, and even the technology and conditions of honey collecting [2,5]. The presence of sugars, phenolic acids, minerals, and amino acids means that honey is a material with a variable molecular structure and nutritional quality [4]. Because many factors affect the composition of honey types, the identification of its origin is not an easy task.

Over the past three decades, numerous scientific studies have been carried out in various parts of the world focusing on the characterization and authentication of honey [1]. Considerable development has been observed in terms of modern technologies enabling the identification of compounds that are only found in a specific type of honey, so-called chemical markers, enabling the determination of the geographic and botanical origins of honeys, even if they are present in small amounts [6]. Furthermore, attempts at assessing botanical or geographic origin are made based on the physicochemical and antioxidative properties of honeys or their chemical composition with the use of multivariate statistics, especially principal component analysis (PCA), linear discriminant analysis (LDA), cluster analysis (CA), and artificial neural networks (ANNs) [2,4,5,7,8]. The modern technologies used include gas chromatography coupled with a flame-ionization detector (GC-FID), gas chromatography coupled with mass spectrometry (GC-MS), high-performance liquid chromatography with photodiode array detection (HPLC-PAD), liquid chromatography-tandem mass spectrometry (LC-MS/MS), high-performance thin layer chromatography (HPTLC), Fourier transform infrared spectroscopy with attenuated total reflectance (FTIR-ATR), nuclear magnetic resonance (NMR), potentiometric tongue, electronic nose, zymography, polymerase chain reaction (PCR), and DNA metabarcoding, among others [1,6].

The objective of this study was a comparative analysis of four honey types from the area of the Carpathian Foothills, located in south-east Poland, based on the content of the main phenolic acids and proline, the mineral composition, and selected physicochemical properties. The honey classification based on the investigated parameters was conducted with the use of multivariate statistical analysis. According to the best of our knowledge, the characterization of honeys from the Carpathian Foothills area based on such parameters has not yet been investigated.

## 2. Results and Discussion

Table 1 presents the mean results of the analysis of the content of selected phenolic acids, proline, and mineral components of the four honey types. The phenolic acid content in the analyzed honey samples varied. Honeydew honeys were characterized by the highest total content of phenolic acids, including chlorogenic acid, whereas caffeic acid was not identified in any sample of this honey type, which may be considered as a specific chemical marker. Buckwheat honeys were found to have the highest content of caffeic acid, with a mean value of 0.520 mg·kg^−1^, and linden honeys the lowest, with a mean content of 0.180 mg·kg^−1^. Ferulic acid was identified in all studied honey types, and the highest concentration of this compound was found in buckwheat and honeydew honeys, at concentrations of 1.320 mg·kg^−1^ and 1.242 mg·kg^−1^, respectively. On the other hand, linden honeys were characterized by the lowest total content of phenolic acids, at a mean level of 2.757 mg·kg^−1^, including ferulic and caffeic acid.

Phenolic compounds constitute a highly diverse group of compounds of plant origin. They are an important group of substances that contribute to the formation of the organoleptic properties of honey, such as color, taste, and smell [9]. In addition, they exhibit antibacterial, antioxidative, and anti-inflammatory properties [5]. Dark-colored honeys, such as buckwheat and heather honeys, are characterized by a higher polyphenol content, and thus their increased antioxidative potential and mineral content than light-colored honeys, such as rapeseed and acacia honey [2,5,8]. The phenolic acids identified in the tested samples are common in honeys, but their concentrations largely depend on the botanical and geographic origin, as well as the climate characteristics of the given region [10]. The analysis of phenolic compounds was also considered a highly promising method of testing the botanical and geographic origin of honeys [9,10,11]. Some phenolic acids, e.g., hydroxycinnamates (caffeic, p-coumaric, and ferulic acids) have also been used as floral markers [12]. What is more, according to Kędzia and Hołderna-Kędzia [13], caffeic, chlorogenic, and ferulic acid are predominant representatives of phenolic acids in Polish honey types, thus in the present study we analyzed their concentrations. Can et al. [14], who examined multifloral honeys, among others, determined amounts of caffeic acid at the level of 2.03 µg·g^−1^, whereas honeydew honeys had a concentration of this substance that was twice as high. On the other hand, a study by Oroian and Sorina [4] confirmed the presence of caffeic acid at the level of 0.02 mg·100 g^−1^ in multifloral honeys and 0.75 mg·100 g^−1^ in honeydew honeys. In the case of ferulic acid, its concentration in the Turkish honeys studied by Can et al. [14] was found to be the highest in multifloral honeys (9.35 µg·g^−1^) and was almost four-fold lower in honeydew honeys. 

An analysis of the proline content in the analyzed honey samples (Table 1) showed the highest concentration of the amino acid in honeydew honey (277.52 mg·kg^−1^) and in buckwheat honey (251.57 mg·kg^−1^), whereas the lowest concentration of proline was found in multifloral honey, being lower on average by 35% than in honeydew honeys.

Proline is the predominant amino acid found in honey (50%–85%). It is mainly formed during the transformation of nectar into honey by bees [15]. Its content is used as the criterion of maturity or adulteration of honey with sugar. The minimum content was established at 180 mg·kg^−1^ [16]. According to a study by Iglesias et al. [17], nectar honeys are characterized by a lower content of amino acids than honeydew honeys. A similar trend was observed in the honey samples analyzed in this study. In Polish honeys analyzed by Kowalski et al. [16] the proline content ranged from 217.17 μg·g^−1^ in rape honey to 389.66 μg·g^−1^ in forest honey. In a study by Janiszewska et al. [15], the mean proline content in buckwheat honey samples was 227.83 mg·kg^−1^, which is similar to the results in our studies concerning this group of honeys. According to Qamer et al. [18], who studied Pakistani nectar honeys, the mean proline content was 276.42 mg·kg^−1^. However, Popek et al. [19] measured an over five-fold lower proline content for Polish nectar honeys as compared with the results obtained by Qamer et al. [18] and a four-fold lower proline content as compared with our honeydew honeys. The above comparison proves that proline content cannot be an unambiguous indicator used in the botanical identification of honey.

Multielemental analysis of the studied honey samples revealed significant concentrations of minerals, particularly in honeydew honeys, which were characterized by the highest K, Mg, Fe, and Cu content, and the sum of all minerals identified was 2408.90 mg·kg^−1^ (Table 1). The lowest content of minerals, including toxic elements, was recorded for buckwheat honeys, at the level of 832.31 mg·kg^−1^. Potassium was the predominant element in all honey types, especially in honeydew honey, with a mean level of 2259.39 mg·kg^−1^. Its concentration in honeydew honeys was almost twice as high as in linden and multifloral honeys, and three times higher than in buckwheat honey. Calcium occupied second place in terms of its content among the analyzed elements, ranging from 38.361 to 83.750 mg·kg^−1^.

Elements constitute a group that is found in honeys in low amounts, but their significance is very high, on the one hand increasing the nutritional value of honey, and on the other being of use as markers of honey types. Micro- and macronutrients mainly originate from nectar, but also from honeydew; thus, their content is determined by the nectar flow from which the honey originates [3]. Honey has a particularly high content of K, as well as Na, P, Mg, and Ca. Slightly lower concentrations are observed for Fe, Si, S, Cu, F, Zn, Mn, and there are trace amounts of Co, I, Mo, Cr, Be, and Ba. Moreover, the elemental content, according to the literature data, is strongly correlated with the color and also the conductivity of honeys [2,6]. The highest concentration of potassium, as compared with the remaining identified mineral components in honey, was demonstrated by Kaygusuz et al. [20], who investigated the mineral content in Turkish honeys. According to the authors of this study, the mean potassium content ranged from 8.53 to 194.79 mg·100 g^−1^, and the highest content of this element was determined in chestnut, pine and oak honey [20]. Potassium is a key element found in honey, and its highest amounts were found, i.a., in multifloral honey from Spain examined by Fernández-Torres et al. [21] and in Italian honeydew honeys analyzed by Pisani et al. [22] (3440 mg·kg^−1^), as well as in multifloral (2782 mg·kg^−1^) and honeydew Croatian honeys (3280 mg·kg^−1^) analyzed by Bilandžić et al. [23]. In Italian multifloral honeys, calcium was present at the level of 254 mg·100 g^−1^, and in honeydew honeys, its content was 356 mg·100 g^−1^ [22,23,24].

Hungarian floral honeys with a light color, analysed by Czipa et al. [25], were distinguished by the highest calcium content (111 mg·kg^−1^) within the group of honeys examined by those authors, similarly to Polish linden honeys with a light color and a Ca content of 83.750 mg·kg^−1^, and this was the highest concentration of this element in the group of Polish honeys analyzed. On the other hand, multifloral honeys and dark honeys such as honeydew and buckwheat honeys were characterized by having close to half of this calcium content at the level of 38.361–58.319 mg·kg^−1^ and were similar to Greek and Spanish citrus honeys, which also had a similar Fe content but close to two-fold higher Mg and Mn contents as compared with the citrus honeys studied by Karabagias et al. [26]. The Mg content in Spanish multifloral honeys was 267.13 mg·100 g^−1^, five-fold higher than in Italian multifloral honeys and three-fold higher than in Portuguese multifloral honeys [22,27].

Honey from Croatia had an Mg content at the level of 69.9–77.7 mg·kg^−1^ in multifloral and honeydew honeys, that is, more than twice that in the Polish honeys we have investigated [23]. However, the Mn content in multifloral honey from Italy was at a similar level of 1.68–1.70 mg·kg^−1^ [22], and it was almost three times lower than in the presently investigated Polish honeys, in which the Mn level was similar to the Turkish honeys analyzed by Yucel and Sultanoglu [28]. Furthermore, Turkish honeys were distinguished by a five-fold higher Zn content as compared with the Polish honeys investigated, and a very low Fe concentration of 10.2 mg·kg^−1^, which was ten times higher than the content of this element in Polish honeys [28]. A similarly high content of iron was recorded in honeydew honeys from Croatia [23] and Italy, and in multifloral honeys it was five times lower and amounted to 2.49 mg·kg^−1^ [22,24]. The content of Zn in honeys studied by the same authors was on average 30%–40% lower than in Polish multifloral and honeydew honeys and amounted to 1.85–1.87 mg·kg^−1^. Honey from the Mediterranean countries were typically characterized by a higher content of mineral compounds as compared with honeys from other parts of the world [22,23,24].

Chudzińska et al. [29], who studied, i.a., mineral components in Polish honeydew honeys, obtained similar results to those of the honeydew honeys we studied in terms of potassium content, whereas the content of Ca, Mg, and Zn in their samples were almost two-fold lower. Furthermore, in terms of the Mn and Cu content, Chudzińska et al. [29] obtained results that were about 20% higher as compared to our honeydew honeys. In terms of toxic metals, the content of Al was 25.7 mg·kg^−1^ and it was lower than our investigated honeydew honeys by about 1/3, the Cd content was 1/3 higher and amounted to 0.041 mg·kg^−1^, and the Pb content of 0.208 mg·kg^−1^ was three times higher than in our study. Buckwheat honeys analyzed by Chudzinska et al. [29] were characterized by a similar range of K, Cu, and Mn content, whereas the levels of calcium at the level of 61.4 mg·kg^−1^ and zinc at the level of 2.28 mg·kg^−1^ were two-fold higher than the content of these elements in the buckwheat honeys we examined. On the other hand, our tested buckwheat honeys stood out, as compared with the honeys studied by Chudzińska et al. [29], having a two-fold higher Mg content of 26.990 mg·kg^−1^, an approx. 1/3 higher Cd content and a three-fold lower Pb content (0.026 mg·kg^−1^), as well as a minor Al content of 3% of the value obtained for buckwheat honeys by Chudzińska et al. [29]. In terms of harmful and toxic elements, the present study revealed the highest Al and Pb content in honeydew honeys and the highest concentration of Cd was found for linden honeys, as well as honeydew honeys.

Aluminum is the third most common element in the Earth’s crust. Aluminum ions may inhibit different metabolic processes due to competitive reactions between Al and other ions, such as Ca, Mg, and Fe. Furthermore, Al exhibits a negative toxicological effect on the central nervous, skeletal and hematopoietic systems [23,30]. With regards to the recommendations set out by international communities and organizations responsible for food safety (the joint FAO/WHO Expert Committee on Food Additives), the provisional tolerable weekly intake (PTWI) of Al without harm to health may amount to 2 mg·kg^−1^ b.w. [31]. In the present study, the Al concentrations in the studied honey types were as follows: honeydew >> multifloral > linden > buckwheat. The content of aluminum in Carpathian multifloral honeys, at a mean level of 4.790 mg·kg^−1^, was substantially lower than that of multifloral Croatian and Turkish honeys (8.52–13.68 mg kg^−1^) [23,28,32]. In turn, the multifloral honeys from Turkey, originating from the Black Sea area, studied by Silici et al. [33], were distinguished by a marginally low Al content (0.0044–0.703 mg·kg^−1^). On the contrary, high Al concentrations were recorded for multifloral honeys from New Zealand [34]; however, this level was lower by almost half in comparison with the studied Carpathian honeydew honeys, for which the mean Al content was 39.020 mg·kg^−1^. In conclusion, honey can be viewed as a reliable biological marker for the assessment of heavy metal pollution.

In terms of cadmium and lead, the PTWI levels are 7 μg and 25 μg·kg^−1^ b.w., respectively [35]. In the context of the aforementioned recommendations, the average daily dose of toxic elements for a human weighing 70 kg consumed in the diet should not exceed 70 µg Cd and 250 µg Pb. Assessing the risk of consumption of the studied honeys from the standpoint of contamination with toxic metals, it can be stated that this risk is minimal, because the consumption of even 1 kg of linden and honeydew honeys, which was found to have the highest Cd concentration, can cover only 40% of the daily intake of this element for a 70 kg person. Studies of other authors revealed high variability in terms of the micronutrient concentration in honeys originating from different regions of Poland, and in particular for Pb with a wide range from 0.007 to 1.21 mg·kg^−1^ [29,36]. As shown by the author’s own research, perhaps low levels of toxic metals do not pose a hazard for adults, but they certainly can raise concerns regarding the provision of such products to children and pregnant women [37]. Exposing pregnant women to high concentrations of lead may result in miscarriage, stillbirth, preterm delivery, and a low birth weight. Alarming reports published by the World Health Organization provide a clear warning that even the lowest concentration of toxic metals, and lead in particular, in food is harmful to small children. Small children are particularly exposed to lead poisoning, because they absorb 4–5 times more of the consumed lead from the same source than adults do; thus, they may incur severe and permanent unfavorable health effects, particularly affecting brain and nervous system development. Lead also results in long-term health damage in adults, including an elevated risk of high blood pressure and renal injury [38]. Minerals, in comparison with other ingredients of honey, such as polyphenols and amino acids, do not undergo degradation under the influence of heating, light, and oxidative and other factors, which affect organic nutrients. This fact is of paramount nutritional importance, because minerals constitute elements of enzymes necessary for a range of metabolic reactions occurring in the human organism, and they play a significant role in systemic function, thus they must be provided through the diet [39]. By analyzing the other elements presented in the studied honeys, it can be determined that replacing sugar with honeydew honey and regular consumption at an average level of 100–150 g (4–7 spoons/day) may markedly enrich the body with potassium, covering an average of 5%–7% of the daily demand for the element, which is known to be a key component ensuring proper water and electrolyte management.

Table 2 presents the sugar profiles and selected physicochemical properties determined for the tested Polish honey types. Based on the quantitative and qualitative chromatographic analysis, no differences between total sugar and carbohydrate content was observed in the studied honeys. Statistical analysis has confirmed highly significant differences between the mono- and oligosaccharide profile in the examined honey samples. The sugars identified in the highest amounts in all of the tested honeys were fructose and glucose. The highest fructose concentration was identified in buckwheat honeys, where its mean content amounted to 48.945 g·100 g^−1^, and the lowest was found for honeydew honeys at the level of 34.585 g·100 g^−1^. The mean glucose concentration was from 31.381 g·100 g^−1^ in multifloral honeys to 28.100 g·100 g^−1^ in honeydew honeys. No statistically significant differences between the studied honeys could be found in terms of glucose content.

Sugars comprise the most numerous group of compounds found in honeys. Their average content is 95%–98%, of which 70%–80% in nectar honeys and 55%–65% in honeydew honeys consist of reducing sugars, i.e., glucose and fructose. Most honeys have a higher content of fructose (approx. 38%) than glucose (approx. 31%) [40]. 

In general, the sugar composition in honeys depends on the variety of flowers used by the bees, as well as the region of the culture and climate conditions [3]. The fructose-to-glucose ratio is a honey-specific trait, which can be used for the purpose of honey classification [6]. Additionally, the ratio between fructose and glucose determines the rate of the honey crystallization process, because glucose is characterized by poorer water solubility than fructose [41]. According to Kaškonienė et al. [42] the higher the F:G ratio, the slower the crystallization process. However, if the glucose content in honey is below 30%, then it will not crystallize at all. Numerous researchers [41,43] have recorded a mean F:G ratio of about 1.2, which overlaps with our results concerning multifloral and honeydew honeys, with 1.260 and 1.269, respectively. On the other hand, Bauer [44] determined more significant differences in the F:G ratio for nectar honeys of 1.0 and honeydew honeys of 1.5–2.0. In the present study, the highest F:G ratios were found in the buckwheat and linden honeys. 

The sugar content has been used as a determinant for the geographic [45] and botanical origin of honeys [43,46]. However, researchers have emphasized that the sugar composition is not a sufficient and homogeneous determinant of honey origin, because chromatographic analyses give very similar results between different honey types [1]. In turn, the present study showed the presence of sugars that could be considered markers for a given honey type, i.e., melezitose, present only in honeydew honeys, and trehalose, identified in the multifloral honey sugar profile.

In a study by Rybak-Chmielewska [47], the mean fructose content in multifloral honeys was approx. 385 mg·g^−1^, whereas in the Bulgarian multifloral honeys studied by Paranov et al. [48], it was about 449 mg·g^−1^. On the other hand, the results obtained by Persano Oddo and Piro [49], who conducted an analysis of honeydew and linden honeys from different regions of Europe, showed that coniferous honeydew honeys had a mean fructose content of 325 mg·g^−1^ and linden honeys had 375 mg·g^−1^, and their results are similar to the results obtained in the present study. However, the coniferous honeydew honeys studied by Rybak-Chmielewska et al. [47] had a fructose content of 315–382 mg·g^−1^, whereas the linden honeys studied by Waś et al. [50] had a mean of 339–406 mg·g^−1^. The fructose content in Spanish honeys was determined at 343–394 mg·g^−1^, in Portuguese honeys it was 314–398 mg·g^−1^, in Italian honeys it was 318–431 mg·g^−1^, in Brazilian honeys it was 278–472 mg·g^−1^, and in Lithuanian honeys it was 329–400 mg·g^−1^ [42].

The glucose content in coniferous honeydew honeys tested by Rybak-Chmielewska et al. [47] was 243–300 mg·g^−1^, and in the linden honeys examined by Waś et al. [50], it was 273–385 mg·g^−1^. On the other hand, a study by Persano Oddo and Piro [49] revealed that the mean value of coniferous honeydew honeys was about 262 mg·g^−1^, and in linden honeys, approx. 319 mg·g^−1^. Paranov et al. [48] determined the glucose content in Bulgarian multifloral honeys at the level of 255 mg·g^−1^. Kaškonienė et al. [42] recorded a glucose concentration in Lithuanian honeys of 346 to 426 mg·g^−1^, also presenting the results of other authors with regard to honey from Spain (258–352 mg·g^−1^), Portugal (274–363 mg·g^−1^), Italy (237–376 mg·g^−1^), and Brazil (240–387 mg·g^−1^).

The minimum content of reducing sugars in nectar honey should be 60 g·100^−1^ [51]. A study carried out by Manzanares et al. [52] determined that nectar honeys had a total sugar content of 82.80% and honeydew honeys had 80.60%. However, Popek et al. [19] found that the total sugar content was 75.00 g·100 g^−1^ in multifloral, 80.90 g·100 g^−1^ in linden, 73.60 g·100 g^−1^ in buckwheat, and 72.19 g·100 g^−1^ in honeydew honeys from Poland.

Disaccharides identified in honeys include sucrose, maltose, cellobiose, isomaltose, maltulose, trehalose, turanose, melibiose, kojibiose, gentiobiose, turanose, laminaribiose, and nigerose [53]. However, their content is highly variable depending on the honey type and it is difficult to indicate a disaccharide that presents a considerable advantage in the identification process [1,3].

The analysis of disaccharides within the studied honeys identified sucrose, the highest content of which was confirmed in honeydew honeys with a mean level of 7.929 g·100 g^−1^, and the lowest in buckwheat honeys at 0.481 g·100 g^−1^. Maltose was not identified in any buckwheat honey sample and the highest amount of this disaccharide was detected in honeydew honeys, with a mean level of 5.072 g·100 g^−1^. Trehalose was only found in multifloral honeys at the level of 0.319 g·100 g^−1^. Among oligosaccharides, the tested honeys were found to contain raffinose at a mean level of 1.036 g·100 g^−1^ in linden honeys and 0.046 g·100 g^−1^ in buckwheat honeys, whereas melezitose was only found in honeydew honeys, at a level of 1.062 g·100 g^−1^ on average.

Sucrose is an important indicator of honey quality; its elevated content may indicate immaturity or honey adulteration, e.g., via feeding bees with sucrose syrup or adding sugar syrups directly to the finished product [3,6]. International standards determine the permissible content of this compound. In general the permissible content is not more than 5 g·100 g^−1^. In false acacia (*Robinia pseudoacacia*), alfalfa (*Medicago sativa*), Menzies Banksia (*Banksia menziesii*), French honeysuckle (*Hedysarum*), red gum (*Eucalyptus camaldulensis*), leatherwood (*Eucryphia lucida*, *Eucryphia milliganii*), and *Citrus* spp. it is not more than 10 g·100 g^−1^. In lavender (*Lavandula* spp.) and borage (*Borago officinalis*) it is not more than 15 g·100 g^−1^ [51]. Melezitose, a trisaccharide, is a characteristic sugar, found only in honeydew honeys. Melezitose is synthesized in aphid and scale insect bodies from sugars found in plant juices. Furthermore, honeydew honeys contain higher levels of other trisaccharides—erlose, raffinose, maltotriose, as well as oligosaccharides [3,54]. Honey authenticity indicators are also sought by determining the proportions of disaccharides, e.g., maltose and isomaltose, or sucrose and turanose. For example, acacia honey is characterized by a high maltose-to-isomaltose ratio, which is considerably lower for linden and honeydew honeys [54].

The relationship between the fructose and glucose and the amount of oligosaccharides in the honey also affects the optical activity, known as specific rotation or optical rotation. In different countries, methods for honeydew and nectar honey quality control require the determination of optical rotation for the analysis of purity according to Council Directive 2001/110/EC [51]. Nectar honeys exhibit negative rotation and honeydew honeys exhibit positive rotation [1]. This is caused by the opposing optical rotation of fructose and glucose/sucrose, which add up, depending on their concentrations. Negative rotation is caused by the predominance of fructose ([α]D20 = 92.4°), whereas positive rotation is caused by that of glucose ([α]D20 = +52.7°), sucrose ([α]D20 = +66.5°), melezitose ([α]D20 = +88.2°), and erlose ([α]D20 = +121.8°) [40]. Thus, optical rotation in honeys is determined by the composition and concentration of the sugars and it is utilized in quality control to determine honeydew honey contamination with floral honeys and vice versa. This relationship was confirmed for the tested honeys. Honeydew honeys were the only ones to be characterized by positive polarity (mean 2.085°), and among the tested nectar honeys, multifloral and buckwheat honeys had the best indices of negative polarity (mean −15.803°; −12.247°). The obtained results agree with the literature data. In a study by Dinkov et al. [55], the mean value of the proper rotation in multifloral honeys from Bulgaria was −14.8°, whereas this value was found to be positive for honeydew honey (+4.2°). A study by Zielińska et al. [40] demonstrated a very similar level of proper rotation in linden honeys to the presented results, which amounted to −7.9°. On the other hand, in a study by Kowalski et al. [56], the determined mean proper rotation for linden honey was −13.97°, buckwheat honey −17.77°, and acacia honey −21.48°, and it was correlated with the content of reducing sugars.

Analysis of the physicochemical properties of the tested species confirmed highly significant differences between the densities of multifloral honey, which were characterized by the highest density, along with honeydew and linden honeys. An excessively low honey density is related to an excessively high water content, and may suggest insufficient maturing time in the combs. This kind of honey is referred to as immature.

The presence of free acids in the honey determines its taste and aroma, as well as its antibacterial properties. Acids are introduced to the honey with nectar, honeydew, pollen grains, and the glandular secretions of bees, and are synthesized in the process of fermentative decomposition and sugar oxidation [3]. The concentration of free acids in the samples of the analyzed types of honey ranged from 8.647 meq acid·kg^−1^ (linden honey) to 20.031 meq acid·kg^−1^ (buckwheat honey). However, substantial variation was observed within the analyzed types. European standards specify the permissible content of free acids, which does not exceed 50 meq acid·kg^−1^ [51]. Free acidity mainly stems from the content of organic acids, as well as inorganic anions, such as phosphate, sulphate, nitrate, and chloride ions [3]. The concentration of free acids depends not only on the botanical origin of the honey, but also on its geographic origin or harvesting season. Elevated acidity may suggest ongoing fermentation [57]. In general, more organic acids are found in dark nectar honeys (e.g., from buckwheat) and honeydew honey [7]. The lowest pH value among the analyzed honeys was determined for buckwheat honey (3.811), and the highest for linden honey (4.907). The nectar honeys were characterized by lower pH (mean 3.9), with the exception of chestnut honey (pH 6), whereas the honeydew honeys had higher values (mean of 4.3) [3]. The pH of honey is not only affected by the compounds responsible for free acidity formation, but also the presence of buffer solutions (salts and acids) and mineral compounds [3]. pH is an important parameter that can be used to analyze honey adulteration [1].

## 3. Chemometric Analysis

Principal component analysis (PCA) was performed to find relationships between variables (physicochemical properties, polyphenolic, mineral compounds, and proline content) and types of honey. Twenty-five variables were reduced to four principal components (PCs). These components explained 83.9% of the variance in the analyzed honey samples (PC1: 43.58%; PC2: 23.03%; PC3: 13.17%; PC4: 4.15%). The obtained results are presented as a projection of PC1 and PC2, because these PCs carry the most information about the examined data set. The most important and first principal component (PC1) was strongly associated with the value of fructose, sucrose, maltose, melezitose, K, Mg, Fe, Al, Pb, chlorogenic acid, and caffeic acid (Table 3). The second component (PC2) could be defined by the levels of Ca, Cu, Mn, ferulic acid, and proline.

**Figure 1:**
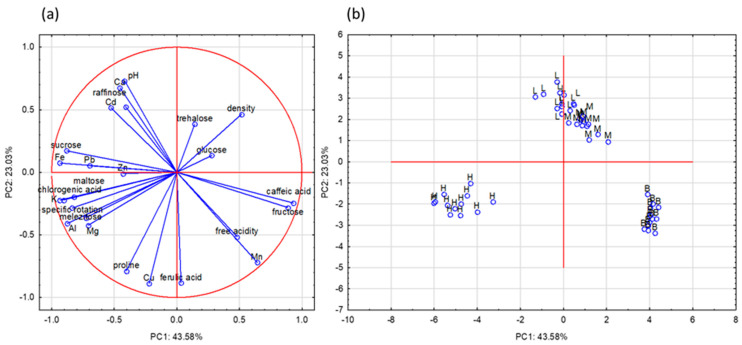
Principal component analysis results: (**a**) projection of chosen variables and (**b**) honey samples as a function of the PC1 vs. PC2; L—lime honey, M—multifloral honey, H—honeydew honey, B—buckwheat honey.

The relationship between the analyzed variables and the studied honey samples is presented in Figure 1. The honeys were classified into three separate groups, only linden and multifloral honeys were not separated, which means that these honeys have similar properties based on the studied parameters. Honeydew honeys are located on the left side of the plot (negative values of PC1 and PC2) and were characterized, among other parameters, by the highest amount of K, Al, Mg, maltose, melezitose, and chlorogenic acid. Another important parameter that distinguished honeydew honey from nectar honey was specific rotation. Buckwheat honeys, concentrated on the right side of the plots, exhibited the highest values of Mn, caffeic acid, fructose, and free acidity compared to the other studied honey samples. Furthermore, linden and multifloral honeys, located in the upper part of the plot, showed high pH values and Ca and Cd contents.

**Figure 2:**
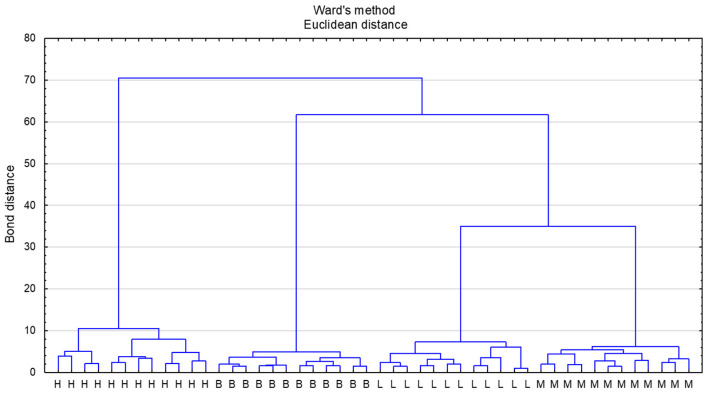
Dendrogram of analyzed honey types according to cluster analysis. Honey type: L—lime honey, M—multifloral honey, H—honeydew honey, B—buckwheat honey.

Cluster analysis (CA) was performed by assuming Euclidean distance as the distance measure and Ward’s method for clustering. The samples with the most similar values of the designated parameters are located closest to each other. The honeys were divided into four main clusters according to the botanical origin declared by the beekeeper (Figure 2). The greatest similarity was observed between linden honey and multifloral honeys, which agrees with the PCA analysis. The close similarity between linden and multifloral honeys based on cluster analysis was also observed by Majewska et al. [57], who differentiated honeys based on physicochemical and antioxidative properties. The most distant cluster comprised honeydew honeys, which stems from their completely different nectar flow, that is, from honeydew and not from nectar. Variable significance was determined based on the C&RT model. Proline was found to be the variable with the highest significance (variable significance = 1). Many variables were assessed as equally significant (fructose, sucrose, maltose, melezitose, Ca, K, Fe, Mn, Al, chlorogenic acid, caffeic acid, specific rotation, and pH—variable significance = 0.90).

**Figure 3:**
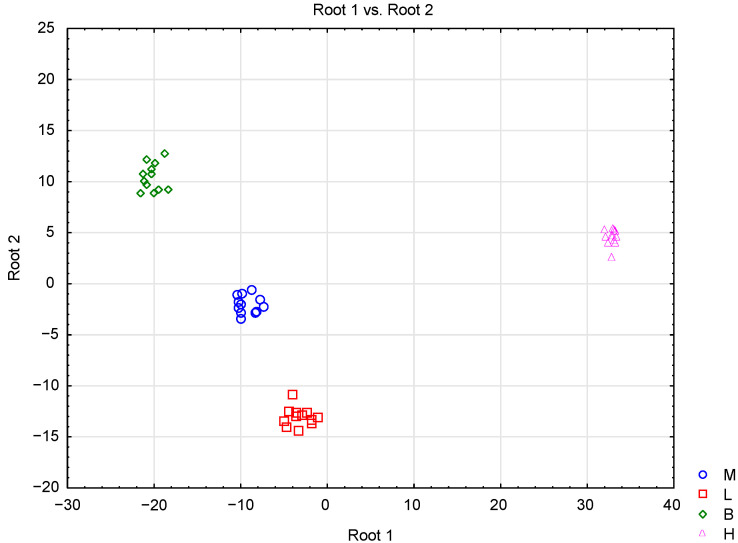
Scatterplot of canonical discriminant scores for analyzed types of honey: M—multifloral honey, L—lime honey, B—buckwheat honey, H—honeydew honey using LDA.

Differentiation of the honey types was also conducted with the use of linear discriminant analysis (LDA). The results showed that three statistically significant (*p* < 0.05) discriminant functions were formed (Wilk’s lambda = 0, χ2 = 467.53, df = 75, *p* = 0) for the first function, (Wilk’s lambda = 0, χ2 = 270.30, df = 48, *p* = 0) for the second, and (Wilk’s lambda = 0.020, χ2 = 126.97, df = 23, *p* = 0) for the third. The first discriminant function accounted for 76.55% of the total variance, the second for 14.62%, and the third for 8.83% (Table 4). Sucrose, trehalose, K, Mg, Mn, chlorogenic acid, specific rotation, and pH contributed the most to the first canonical variable. The second canonical variable was related to fructose, Ca, Mn, and ferulic acid, whereas the third was related to the sucrose, Ca, and proline contents. This means that these variables were the most important in discriminating between honey samples (the higher the absolute value of the standardized coefficient, the more important was the related independent variable). According to the classification matrix, all types of honey were classified correctly (Table 5). A scatter plot of canonical values (Figure 3) shows that the honey samples were clearly separated based on their botanical origin, by the two first discriminant functions. It is noticeable that nectar honeys were positioned at the left side of the scatterplot, whereas honeydew honeys were positioned at the right side. Honeydew honeys were the most separate group, which confirms previous observations obtained by PCA and CA methods.

All the statistical methods were used to differentiate honey types based on the analyzed parameters. Many studies have proven that multivariate statistical analysis can be used for differentiating honey samples based on physicochemical properties and the content of biologically active compounds [2,4,7,8,26]. Certain studies only used physicochemical parameters to differentiate honeys [19,57] or the content of specific compounds, e.g., mineral compound profile [29], amino acids [15,17], phenolic compound profile [10], or antioxidant activity [5]. Honey classification is mainly performed based on the biological origin of the samples, and less often on the geographical one. Multivariate statistical analysis can also be used to detect adulteration in honey, and to indicate poor-quality honeys [58].

## 4. Materials and Methods

### 4.1. Honey Samples

**Figure 4:**
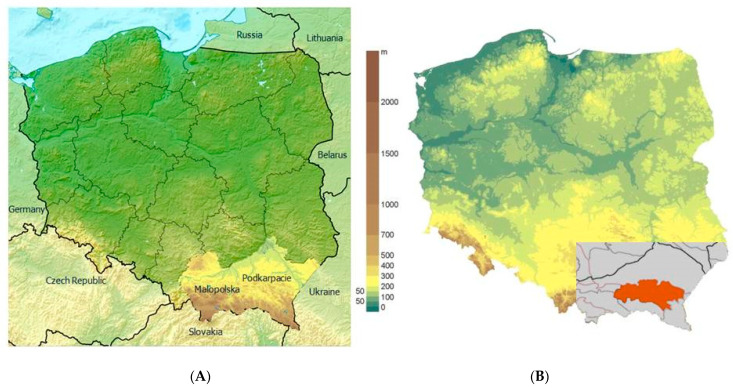
Map of Poland with Malopolska and Podkarpacie provinces (**A**) and with the regions where honey samples were collected (**B**).

The study material comprised 48 honey samples from apiaries located in mountainous areas within the Carpathian Foothills region in south-eastern Poland. Honeys originating from clean areas of the Carpathian Foothills with low industrial development, around the border between two voivodeships, were selected for the study. The Carpathian Foothills is the lowest portion of the Polish Carpathians, forming a group of low-altitude hills between 350 and 600 m a.s.l., with smooth and round slopes. The Carpathian Foothills are characterized by moderate, intermediate, and submontane climates. Below is a map of the locations of the apiaries from which the tested honey samples originated (Figure 4). Four types of honey were analyzed (12 samples from each type): multifloral (spring), linden, buckwheat, and honeydew (pine) (Table 6). The honey was kept in a dark place at room temperature (21 °C ± 1 °C) until analysis. The floral origin of the samples was specified by the beekeepers according to the hive location and available floral sources.

### 4.2. Analytical Procedures

#### 4.2.1. Profiles of Phenolic Compounds

The contents of phenolic acids were determined according to the method described by Can et al. [14] with modifications. A weighed honey sample of 20 g was dissolved in 100 mL of acidified distilled water in a conical flask at room temperature. The solution was mixed in a magnetic agitator until complete dissolution was obtained, and then filtered through a paper filter on a filter under reduced pressure. Clear honey solutions were applied on conditioned media of an SPE 16- or 24-Port SPE Vacuum Manifolds filtration set from Thermo Scientific™ (Waltham, MA USA) (C18 500 mg medium). Polyphenols were leached from the columns with methanol directly in a round-bottomed flask and were concentrated at 40 °C until the solvent was evaporated in a Hei-VAP Precision rotational vacuum evaporator from Heidolph (Schwabach, Germany). The honey extracts in the round-bottomed flasks were dissolved with 50% hydrated acetonitrile and filtered through PTFE socket filters with a 0.45 µm pore size directly prior to chromatographic analysis. Analysis of the phenolic acid content was performed with the use of a Thermo Dionex Ultimate 3000 high-performance liquid chromatograph with a UV detector and a DAD-3000 (RS) diode matrix (ESA, Chlemsford, MA USA). Chromatographic separation was conducted using an RP C18 Luna 150 mm × 4.6 mm ID × 5 μm column from Phenomenex at room temperature. UV/DAD detection was carried out at 290 nm and 340 nm wavelengths. The injection volume was 10 µL, and analysis time was 65 min, with variable concentration gradient of solvents—A: 5% formic acid; B: methanol/acetonitrile 2:1 (*v*/*v*). The mean recovery for the honey solutions was 98%. The operation of the chromatographic set and processing of the obtained data were coordinated using Chromeleon 7.2 software (Dionex (Sunnyvale, CA, USA)).

#### 4.2.2. Proline Content

The proline concentration was determined using the method described by Tarapatskyy et al. [59] with their own modifications. The honey solutions were filtered using Syringe filter Sep-Pak C18 Cartridges from Waters (Ireland), and after the evaporation of the extracts in a vacuum evaporator at 40 °C, the residues in round-bottomed flasks were dissolved using sample dilution buffer with pH = 2.2, diluted 4 times, and filtered using a socket filter with a pore size of 0.22 µm directly prior to analysis. The Sykam S433 Amino acid analyzer consisted of a specialized HPLC device made of a cooled reagent chamber with an S7130 degasser, San 5200 autosampler with cooling, an S4300 reaction chamber, a set of columns for physiological amino acid analysis composed of amine precolumn (100 mm × 4.6 mm), and a separation cation exchange column (150 mm × 4.6 mm). A physiological reagent kit containing A-Li-citrate buffer with pH = 2.9, B- Li-citrate buffer with pH = 4.2, and C-Licitrate/borate buffer with pH = 8.0 were used for separation, and regeneration solution was utilized to regenerate the column after amino acid separation using ninhydrin reagent. All reagents were placed in a cooled chamber and stored in an inert gas (argon) atmosphere. The injection volume was 10 µL. Amino acid separation was conducted in a gradient at 80 °C, and the post-column derivatization was performed in the reaction chamber with a ninhydrin contribution at 130 °C. The analysis time was 110 min. Amino acid detection was performed using a UV detector at two wavelengths: 440 and 570 nm. The system stability was controlled with injections of amino acid mixture standard. The amino acid separation system was coordinated using Clarity software from DataApex.

#### 4.2.3. Mineral Content

The concentrations of Ca, K, Mg, Fe, Cu, Mn, Zn, Al, Cd, and Pb were determined by means of inductively coupled plasma optical emission spectrometry (ICP-OES) using a Thermo iCAP 6500 spectrophotometer (Thermo Fisher Scientific Inc., Waltham, MA USA) described by Dżugan et al. [60].

#### 4.2.4. Sugar Profile

The sugar characteristics of the honeys were assessed according to the method described by Dżugan et al. [61]. A Thermo Dionex Ultimate 3000 high performance liquid chromatograph with a Corona Veo RS detector (ESA, Chlemsford, MA USA) was used. The mean recovery for the sugars in the honeys was 90%–98%. The precision of the described analytical method was confirmed by repeating the injections of the standard and each sample three times. The stability of the chromatography system was controlled at four-hour intervals by means of injections of selected standard solutions with known concentrations.

#### 4.2.5. Physicochemical Properties

Physicochemical parameters such as pH, free acidity, and specific rotation were determined according to the standardized methods [62]. pH was measured at 20 °C in a 20% (*w*/*v*) honey solution in ultrapure water using a pH-meter (Mettler Toledo, Warsaw, Poland). Free acidity was determined using the titrimetric method. The honey solution in ultrapure water (20% *w*/*v*) was titrated using 0.1 M NaOH, up to pH 8.3 and the results are expressed as meq acid·kg^−1^. Specific rotation was determined by means of the polarimetric method. Five milliliters of Carrez I solution and Carrez II solution were added to the 6 g of honey dissolved in ultrapure water, which was then topped up to a volume of 50 mL volumetric flask with ultrapure water and left for 24 h. Then the samples were filtered through a filter paper and the obtained solutions were analyzed on a circular polarimeter (P1000-LED, Kruss Optronics, Hamburg, Germany). Specific rotation was calculated using Biot’s formula. Density was measured using a DDM 2910 automatic Density Meter, with high-precision temperature control of the sample. Calibration was performed with air and distilled water.

### 4.3. Statistical Analysis

All of the analyses were performed in three independent replications for each honey sample. The acquired findings were subjected to statistical analyses with the use of Statistica ver. 13.1 (StatSoft, Inc., Tulsa, OK, USA). Significant differences between types of honey based on tested parameters were obtained through a one-way analysis of variance (ANOVA), followed by Duncan’s multiple range test. In order to indicate the relationship between the investigated variables and the analyzed samples, a multivariate statistical analysis was performed, using principal component analysis (PCA), cluster analysis (CA), and linear discriminant analysis (LDA).

### 4.4. Chemicals and Reagents

For the purpose of determinations, analytical purity reagents (analytical standards) designed for liquid chromatography were used: hydrochloric acid, formic acid, ethanol, and acetonitrile from Sigma Aldrich (Steinheim, Germany), and methanol from J.T. Baker Mallinckrodt Baker B.V. Holland. Buffers, ninhydrin, and a mixture of standards for amino acid identification were obtained from Sykam (Eresing, Germany), standardized for amino acid analyses in the physiological (native) range. Analytical standards of chlorogenic, caffeic, and ferulic acid for HPLC were obtained from Extrasynthese (Genay, France). Analytical standards D-(+)-glucose, D-(−)-fructose, D-(+)- sucrose, D-(+)- melezitose, D-(+)-turanose, D-(+)-trehalose, D-(+)-raffinose BioXtra, Ca, K, Mg, Fe, Cu, Mn, Zn, Al, Cd, and Pb were obtained from Sigma- Aldrich (Steinheim, Germany), and D-(+)-maltose standard was obtained from Toronto Research Chemicals. Carrez I solution, Carrez II solution (Sigma Aldrich, Steinheim, Germany), and deionised water from a deioniser from Hydrolab Polska HLP 5P were used.

## 5. Conclusions

Honey has valuable nutritional, therapeutic, and prophylactic properties, which result from its chemical composition. A detailed analysis of the results of comprehensive physicochemical research on the selected types of Polish honey showed that multifloral honeys exhibit almost a two-times-higher content of phenolic acids compared to linden honey, but half the amount compared to honeydew honey; furthermore, caffeic acid was not identified in any honeydew honey. Honeydew honeys were the richest in phenolic acids and minerals, as well as oligosaccharides, of all the studied honey types. Dark-colored honeys were characterized by the highest proline content. The dominant elements in all types of honey were potassium and calcium. The analyzed honeys also contained a significant amount of magnesium. Additionally, honeydew honeys contained the highest amount of toxic metals.

The results of the present study showed that the specific phenolic acids, minerals, proline, and sugar content, in combination with chemometrics analysis, may successfully differentiate between the biological origins of honey samples and allow the preliminary verification of samples before performing time-consuming pollen analysis.

## Figures and Tables

**Table 1:** Comparison of phenolic acids, proline, and mineral content of different types of honey (mg·kg^−1^).

Component (Min.; Max.)	Multifloral	Linden	Buckwheat	Honeydew
Chlorogenic acid	3.240 ^BGI^ (2.26; 3.84)	2.040 ^DHa^ (1.46; 2.49)	1.130 ^FJb^ (0.88; 1.43)	6.500 ^ACE^ (5.50; 7.44)
Caffeic acid	0.250 ^Ba^ (0.14; 0.34)	0.180 ^Db^ (0.11; 0.28)	0.520 ^AC^ (0.44; 0.62)	N/D -
Ferulic acid	0.940 ^BbG^ (0.79; 1.11)	0.530 ^DFH^ (0.43; 0.62)	1.320 ^AC^ (1.08; 1.78)	1.242 ^aE^ (1.10; 1.40)
**Total identified phenolic acids**	**4.434 ^BGI^** **(3.28; 5.28)**	**2.757 ^FJL^** **(2.19; 3.35)**	**2.967 ^DHK^** **(2.42; 3.58)**	**7.743 ^ACE^** **(6.87; 8.77)**
Proline	184.44 ^FJL^ (172.15; 194.23)	214.65 ^DHK^ (201.30; 228.16)	251.57 ^BGI^ (240.33; 261.49)	277.52 ^ACE^ (263.65; 288.65)
Ca	50.080 ^DI^ (42.91; 64.36)	83.750 ^ACE^ (82.08; 85.61)	38.361 ^FHJ^ (35.10; 41.04)	58.319 ^BG^ (51.27; 68.43)
K	948.59 ^Bb^ (648.11; 1222.46)	1231.95 ^DaG^ (1062.91; 1453.32)	755.41 ^FH^ (650.23; 895.43)	2259.39 ^ACE^ (2094.21; 2476.67)
Mg	32.190 ^BgI^ (28.18; 39.29)	24.170 ^DJ^ (20.92; 26.91)	26.990 ^Fh^ (23.98; 31.08)	41.960 ^ACE^ (38.01; 47.22)
Fe	0.790 ^DH^ (0.52; 1.12)	1.470 ^BGI^ (1.22; 1.68)	0.360 ^FJ^ (0.26; 0.42)	2.150 ^ACE^ (1.12; 2;60)
Cu	0.410 ^DH^ (0.33; 0.48)	0.460 ^BF^ (0.38; 0.57)	0.890 ^EG^ (0.60; 1.22)	0.960 ^AC^ (0.79; 1.15)
Mn	4.110 ^BG^ (3.70; 4.71)	2.10 ^FHJ^ (1.96; 2.32)	9.010 ^ACE^ (7.44; 11.03)	3.650 ^DI^ (3.31; 4.02)
Zn	2.660 ^AC^ (1.43; 3.40)	0.790 ^DH^ (0.16; 1.52)	0.900 ^BF^ (0.56; 1.24)	2.350 ^EG^ (2.18; 2.52)
Al	4.790 ^B^ (2.17; 8.21)	2.050 ^D^ (1.63; 2.46)	0.350 ^F^ (0.25; 0.47)	39.020 ^ACE^ (29.82; 48.77)
Cd	0.030 ^E^ (0.02; 0.05)	0.031 ^A^ (0.02; 0.04)	0.011 ^BDF^ (0.01; 0.02)	0.030 ^C^ (0.02; 0.04)
Pb	0.058 ^E^ (0.04; 0.08)	0.044 ^B^ (0.02; 0.09)	0.026 ^DF^ (0.02; 0.04)	0.073 ^AC^ (0.05; 0.10)
**Total identified minerals**	**1043.70 ^DbI^** **(729.76; 1342.03)**	**1346.82 ^BaG^** **(1178.19; 1570.51)**	**832.31 ^FHJ^** **(725.23; 971.26)**	**2408.90 ^ACE^** **(2226.71; 2631.35)**

Results are expressed as a mean value obtained for each honey type and min-max values; N/D—not detected. Statistically significant differences between means (^A–L^ for *p* ≤ 0.01; ^a–b^ for *p* ≤ 0.05) are marked by different letters in the rows.

**Table 2:** Comparison of sugar composition and some physicochemical properties of different types of honey.

Component (Min.; Max.)	Multifloral	Linden	Buckwheat	Honeydew
Fructose (g·100 g^−1^)	39.548 ^BGI^ (36.72; 42.71)	39.423 ^DHK^ (37.75; 41.40)	48.945 ^ACE^ (46.49; 52.50)	34.585 ^FJL^ (32.56; 36.72)
Glucose (g·100 g^−1^)	31.381 (29.89; 32.69)	28.265 (26.10; 30.10)	29.543 (26.55; 31.24)	28.100 (20.12; 33.87)
Sucrose (g·100 g^−1^)	5.399 ^BG^ (4.02; 6.78)	4.113 ^DI^ (3.22; 5.08)	0.481 ^FHJ^ (0.35; 0.67)	7.929 ^ACE^ (5.17; 10.46)
Maltose (g·100 g^−1^)	1.012 ^D^ (0.34; 1.71)	1.398 ^B^ (0.60; 2.71)	N/D -	5.072 ^AC^ (1.48; 10.65)
Trehalose (g·100 g^−1^)	0.319 (0.14; 0.50)	N/D -	N/D -	N/D -
Melezitose (g·100 g^−1^)	N/D -	N/D -	N/D -	1.062 (0.13; 2.45)
Raffinose (g·100 g^−1^)	0.610 (0.27; 1.45)	1.036 ^a^ (0.07; 2.64)	0.046 ^b^ (0.02; 0.08)	0.653 (0.17; 1.11)
**Total identified sugars** **(g·100 g^−1^)**	**78.268** **(73.74; 81.52)**	**74.235** **(70.58; 78.46)**	**79.016** **(73.99; 83.63)**	**77.403** **(69.55; 86.77)**
F/G	1.260 ^B^ (1.12; 1.38)	1.398 ^b^ (1.29; 1.49)	1.666 ^aAC^ (1.49; 1.77)	1.269 ^D^ (1.08; 1.77)
Density (kg·m^−3^)	1.418 ^A^ (1.414; 1.422)	1.416 ^C^ (1.410; 1.423)	1.415 ^a^ (1.411; 1.422)	1.410 ^BDb^ (1.403; 1.416)
Specific rotation (°)	−15.803 ^FJL^ (−17.08; −14.98)	−6.903 ^BGI^ (−8.07; −5.47)	−12.247 ^DHK^ (−14.19; −10.32)	2.085 ^ACE^ (0.66; 4.16)
pH	4.647 ^C^ (3.97; 5.27)	4.907 ^A^ (4.67; 5.26)	3.811 ^BDF^ (3.71; 3.90)	4.484 ^E^ (4.18; 4.84)
Free Acidity (meq acid·kg^−1^)	18.751 ^Ea^ (15.66; 22.98)	8.647 ^DFd^ (4.78; 12.62)	20.031 ^AC^ (16.83; 23.11)	13.977 ^Bbc^ (10.79; 19.81)

Results are expressed as a mean value obtained for each honey type and min-max values, N/D—not detected. Statistically significant differences between means (^A–L^ for *p* ≤ 0.01; ^a–b^ for *p* ≤ 0.05) are marked by different letters in the rows.

**Table 3:** Loadings of the variables for the four principal components.

Variables	PC1	PC2	PC3	PC4
Fructose	0.888	−0.281	0.188	−0.095
Glucose	0.280	0.135	−0.473	−0.674
Sucrose	−0.882	0.172	−0.321	−0.094
Maltose	−0.824	−0.196	0.064	0.188
Trehalose	0.143	0.385	−0.830	0.179
Melezitose	−0.723	−0.362	−0.043	−0.214
Raffinose	−0.410	0.525	0.185	0.424
Ca	−0.455	0.675	0.506	0.016
K	−0.934	−0.222	0.089	−0.027
Mg	−0.709	−0.442	−0.436	0.191
Fe	−0.934	0.074	0.205	−0.037
Cu	−0.221	−0.889	0.199	0.037
Mn	0.642	−0.720	−0.040	0.080
Zn	−0.432	−0.009	−0.813	0.092
Al	−0.875	−0.409	−0.125	−0.018
Cd	−0.528	0.516	−0.249	−0.284
Pb	−0.696	0.053	−0.259	0.140
Chlorogenic acid	−0.903	−0.220	−0.278	0.040
Caffeic acid	0.933	−0.246	0.037	0.081
Ferulic acid	0.034	−0.884	−0.296	−0.086
Proline	−0.404	−0.787	0.365	−0.112
Density	0.516	0.464	−0.242	0.094
Specific rotation	−0.837	−0.285	0.395	−0.131
pH	−0.417	0.731	0.007	−0.235
Free acidity	0.482	−0.514	−0.586	0.117

**Table 4:** Standardized canonical discriminant function coefficients.

Variables	Root 1	Root 2	Root 3
Fructose	−0.019	0.738	0.194
Glucose	0.012	−0.462	0.112
Sucrose	0.823	0.229	−0.923
Maltose	−0.256	0.354	−0.315
Trehalose	−1.022	0.371	−0.408
Melezitose	−0.202	0.231	0.181
Raffinose	0.351	−0.196	0.274
Ca	−0.140	−0.992	0.880
K	0.715	0.253	−0.397
Mg	1.177	−0.303	−0.055
Fe	0.286	−0.259	0.163
Cu	0.456	−0.126	0.373
Mn	−0.785	0.805	−0.230
Zn	0.431	0.170	−0.585
Al	0.103	0.283	0.402
Cd	−0.099	−0.308	0.385
Pb	−0.277	0.211	−0.525
Chlorogenic acid	1.096	−0.159	−0.210
Caffeic acid	0.011	−0.175	0.251
Ferulic acid	0.297	0.711	−0.155
Proline	0.255	0.062	0.704
Density	−0.421	0.219	−0.307
Specific rotation	0.626	0.486	0.188
pH	1.089	−0.588	−0.073
Free acidity	0.134	−0.173	0.170
Eigenvalue	431.81	82.48	49.81
Discrimination (%)	76.55	14.62	8.83
Cumulative (%)	76.55	91.17	100.00

**Table 5:** Classification results of the analyzed honeys using the LDA.

**Original Group**	**Predicted Classification (Number of Samples)**				**Correct Classification [%]**
	Multifloral	Lime	Buckwheat	Honeydew	
Multifloral	12	0	0	0	100
Lime	0	12	0	0	100
Buckwheat	0	0	12	0	100
Honeydew	0	0	0	12	100
Total	12	12	12	12	100

**Table 6:** The characteristics of honey samples.

Category	Type of Honey	Origin	Color	Aroma/Flavor	Location
light	Multifloral	Nectar	light golden, dark yellow to amber	very sweet aroma and mild taste	Podkarpacie
	Linden	Nectar		spicy with light bitterness taste	Malopolska
dark	Buckwheat	Nectar	dark-tea, brown to black	distinctive aroma and spicy taste	Maloposka
	Pine Honeydew	Honeydew		delicate sweet, slightly spicy, resinous taste	Podkarpacie

## Data Availability

Not applicable.

## Sample Availability

Samples of the compounds are not available from the authors.
